# Supplementary material for: lncRNA 1700101O22Rik and NONMMUG030480.1 Are Not Essential for Spermatogenesis in Mice
Source: Int J Mol Sci. 2022 Aug 3;23(15):8627. doi: 10.3390/ijms23158627 (PMC9369125; doi:10.3390/ijms23158627)
Supplement: Supplementary file 1 [file ijms-23-08627-s001.zip › ijms-1801050-supplementary.pdf]

Supplementary Materials

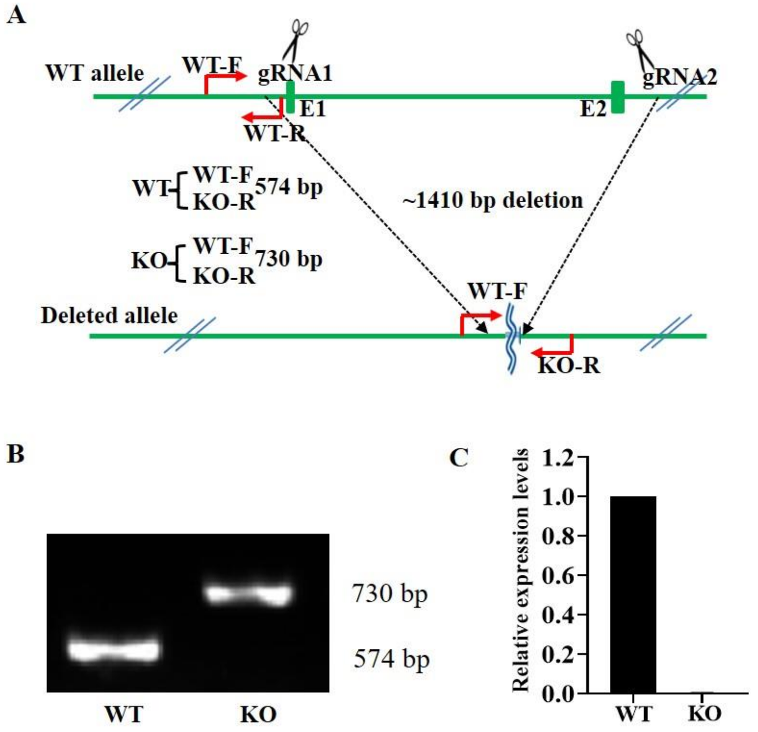

Figure S1. Generation and confirmation of NM480 knockout (KO) mice. (A) A schematic illustration of the Clustered Regularly Interspaced Short Palindromic Repeat (CRISPR)-Cas9-produced NM480 KO mice. (B) Identification of the genotypes in WT and KO mice. 730 bp bands were recognized in KO mice and 574 bp bands were detected in WT mice. (C) Validation of KO mice by analyzing the transcript expression of NM480 with qPCR.

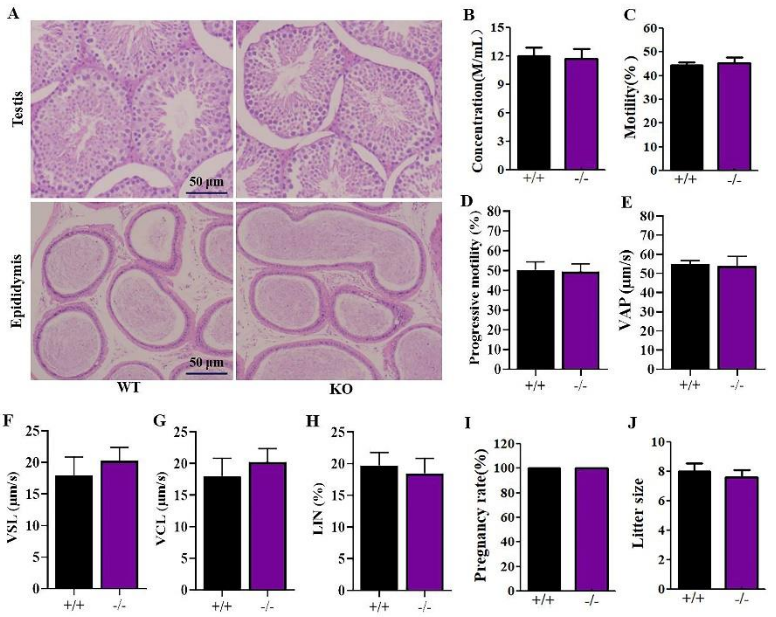

Figure S2. Reproductive phenotype analysis in NM480 knockout (KO) mice. (A) Histological analyses of testis and epididymis sections from NM480 WT and KO mice by haematoxylin+eosin staining. (B) Sperm concentration, (C) total motility, (D) progressive motility, (E) VAP, (F) VSL, (G) VCL and (H) LIN were measured by a computer assisted sperm analysis system. (I) Pregnancy rate and (J) litter size was tested by following mating of male KO and WT mice with proven fertile female mice over a period of 3 months. Data are the mean ± SEM (n = 3).
